# School bullying among migrant children in China: A cross-sectional study

**DOI:** 10.3389/fpsyg.2022.1027506

**Published:** 2022-12-14

**Authors:** Zhengmin Yang, Ying Tu, Zaihua Qin, Xiaoqun Liu, Dali Lu

**Affiliations:** ^1^Department of Pediatric Psychology, Shenzhen Longhua Maternity and Child Healthcare Hospital, Shenzhen, China; ^2^Department of Maternal and Child Health, Xiangya School of Public Health, Central South University, Changsha, China; ^3^Department of Student Affairs, Hunan University of Finance and Economics, Changsha, China

**Keywords:** migrant children, school bullying, prevalence, influencing factors, China

## Abstract

**Background:**

Bullying is a serious public health concern affecting the physical and mental health of children. Migrant children are at higher risk of developing health problems. We conducted this study to investigate the prevalence of school bullying and its possible influencing factors of migrant children.

**Methods:**

A cross-sectional study was carried out in Hunan Province, China from April to July 2018. Multi-stage cluster sampling was adopted to achieve a representative sample covering both urban and rural areas. Migrant children are defined as those who migrate with one or both parents to other places and who do not have a hukou in their city of residence. The Chinese version of Olweus Bully/Victim Questionnaire was applied to measure children’s involvement in school bullying.

**Results:**

A total of 7,607 students were surveyed, including 995 migrant children and 6,612 non-migrant children. The prevalence of school bullying was significantly higher in migrant children than in non-migrant children (*χ*^2^ = 22.740; *p* < 0.001). Binary regression analysis showed that male, middle school identity, more times of playing violent games, more social friends owning and being beaten by parents or caregivers may increase the risk of involvement of school bullying in migrant children.

**Conclusion:**

Migrant children showed a higher prevalence of school bullying than non-migrant children. Gender, grade, frequency of playing violent games, number of social friends and being beaten by parents or caregivers were associated with school bullying in migrant children.

## Introduction

School bullying is a special kind of aggressive behavior, which has the characteristics of deliberate, repetitive, long-term and power imbalance. School bullying was always divided into traditional bullying and cyberbullying, and traditional bullying can be divided into physical, verbal and relational bullying (Gradinger et al., 2009). In addition, school bullying usually involves being bullied and bullying others, that is bullying victimization and bullying perpetration. A survey among school-age children in America showed that the reported rate of physical bullying perpetration (Sujung and Min, 2018), verbal bullying perpetration and relational bullying perpetration was 10.7, 25.2, and 17.5% respectively; the rate of physical bullying victimization, verbal bullying victimization and relational bullying victimization was 13.3, 36.8, 34.9%, respectively. And in mainland China, several national sample studies have shown prevalence of self-reported bullying victimization and perpetration ranged from 26.1–52.1 and 9–34.6%, respectively (Han et al., 2017; Xu et al., 2022). The large number of studies mentioned above show that school bullying is prevalent worldwide.

As one of the global public health events, bullying has seriously affected the physical and mental health development of young people. There is much evidence show that school bullying will lead to a series of problems for those children involved in bullying, such as anxiety and depression, and even including non-suicidal self-injury and suicide in severe cases (Wang et al., 2015; Guo et al., 2019; Martínez-Monteagudo et al., 2020). Longitudinal studies have also shown that victims of bullying in early childhood and adolescence have a strong negative impact on later social and psychological outcomes (Takizawa et al., 2014; Harris and Chun, 2020). For example, bullying perpetration increased the risk of later violence by about two-thirds (Fisher et al., 2012), and frequent peer bullying during childhood increased the risk of intentional self-harm as a teenager (Takizawa et al., 2014). Therefore, it is important to focus on school bullying for the development of the physical and mental health of adolescents.

Previous studies found that rural children, single parents, migrant and left-behind children are more likely to suffer from all types of school bullying (Ttofi et al., 2012). However, most of the current studies on school bullying are conducted in the general population to investigate the occurrence of school bullying and its influencing factors, migrant is only investigated as one of the influencing factors, and few studies focused on the occurrence of school bullying in migrant children. Nevertheless, migrant children tend to perceive less family support and community support than their peers, which indicates more bullying behaviors (Li and Hu, 2020). So it is necessary to pay attention to the bullying behaviors of migrant children.

The term “migrant children” in China refers to school-age children who are registered in other provinces (regions, cities) or counties (districts) outside the province, and go to the import place with their parents (live together) and receive education at school. According to statistics from the Education department, approximately 14.24 million migrant children were in compulsory education in 2018 (Cui and Siu-ming, 2019). To best of our knowledge, due to the impact of China’s household registration or hukou system, as well as the change of region, culture and living environment brought by migration, migrant children often faced a lot of problems, which will not only bring about some mental health problems for them (Ministry of Education of the People's Republic of China, 2019), but also contribute to some adaptive problems, such as experiencing exclusion, discrimination and peer victimization, and engaging in bullying perpetration (Hou et al., 2011; Li and Jiang, 2018; Shi, 2018; Zhang et al., 2019; Li and Hu, 2020) etc. These all made it harder for them to integrate into the group in the new migration environment.

Despite there is evidence of how immigration and bullying are related in China, certain limitations in that relationship still requires scholarly attention. Firstly, the bullying questionnaire used in previous studies (Ye et al., 2016; Li and Hu, 2020) on migrant children is not widely used in China. Since different bullying questionnaires may lead to uneven final results, it is necessary to use bullying questionnaires with good reliability and validity in the specific context of China. Secondly, the selection of migrant children in some of past studies (Li and Jiang, 2018; Zhang et al., 2019; Cui and To, 2021) was limited to urban schools, and did not include rural schools. In fact, migrant children do not only have rural-to-urban and urban-to-urban migration, but may also have rural-to-rural and urban-to-rural migration. On this account, in cases where the background and identity of students are relatively uniform, this may weaken the particularity of migrant identity and cannot truly reflect the situation faced by migrant children. For good understanding of the situation of bullying among migrant children in the complex and more common background, it will be particularly important to integrate migrant children into ordinary schools. Thirdly, migrant children will adjust their cognition and behavior as they are faced with a different living environment, school and friendship from that they are familiar with before, migrant status itself may not directly lead to migrant children being involved in bullying (Li and Hu, 2020). Hence, in addition to further study the relationship between migration and bullying under the special cultural background of China, exploring some potential factors affecting school bullying of Chinese migrant children is also significant.

The social ecological theoretical framework proposed by Professor Bronfenbrenner (Zhu and Yang, 2019) well explained the importance of environment for the analysis and understanding of human behavior early in the 1970s. According to this theory, the social ecological factors affecting school bullying can be roughly divided into four parts, including individual factors, family factors, school factors and social factors. Previous studies have shown that individual demographic variables such as grade and gender of migrant children affect their social adaptation and the education level of parents can compensate for the health difference between migrant children and general children (Bronfenbrenner, 1977). Wang et al. (2016) found that the higher the family economic income, the higher the degree of urban integration of migrant children. In another study, sense of school belonging can buffer the effects of bullying on the mental health of migrant children (Wang et al., 2010). These studies suggest that many factors, such as individual characteristics, family and school factors, will affect the mental health of migrant children, which is in line with the interpretation of ecosystem theory, but most of the existing studies focus on some aspects, and there is a lack of comprehensive research on the factors affecting the school bullying of migrant children from the perspective of ecosystem theory. In view of the complexity of school bullying, especially among migrant children, this study takes ecosystem theory as the framework to explore the risk and protective factors of bullying among migrant children. Firstly, based on the existing research, we include some common variables that may influence school bullying (e.g., gender, grade, academic performance, family economic status, mother education level, school location and so on) into our study. At the same time, considering the particularity of migrant children’s life experience, this study also pays special attention to the migrant children’s experience of transferring school and the number of social friends. As we know, migrant children will experience once or more school transferring, which means leaving the familiar environment to adapt to the new school, teachers and friends relationship. In addition, migrant children’s parents will be busy making a living and have no time to monitor their children, so that migrant children will make more social friends to gain a sense of belonging. So far, it is unclear whether school transferring experience and the number of social friends will be related to bullying of migrant children.

In conclusion, the main purpose of this study is to investigate the prevalence of school bullying among migrant children and non-migrant children, and to initially explore its possible influencing factors among migrant children. We compared the personal characteristics, their family factors and school factors of migrant children who are involved in bullying and those who are not involved in bullying, trying to find some potential differences. This study is the first one in China to explore the influential factors of school bullying among migrant children based on social ecology theory. Given the importance and serious consequences of migrant children’s engagement of school bullying, this study will contribute to a clearer understanding of the influencing factors of school bullying among migrant children, and finally provide targeted theoretical basis for the prevention and intervention of school bullying in migrant children.

## Materials and methods

### Study design and participants

This study was a cross-sectional survey conducted from April to July 2018. The participants were recruited from Hunan Province, where the floating population was 6.86 million, accounting for 10.44% of the permanent population (Nie et al., 2022). It provided great convenience for us to carry out research on migrant children. We used a geography-based stratified cluster sampling frame, which included four cities selected randomly from eastern, southern, western and northern parts of the province, respectively. Schools in each prefecture-level city are divided into urban regions and rural regions. Two junior high schools and two senior high schools were selected randomly from each chosen region, and six classes were randomly selected from each school. Migrant children are defined as those who migrate with one or both parents to other places and who do not have a hukou in their city of residence. Questionnaires were sent out to 8,439 students from 32 sampled schools; of these, 7,607 (90.1%) completed the survey without apparent logical errors and missing items in the questionnaire.

The study received the approval from the ethical committee of Xiangya School of Public Health, Central South University (XYGW-2017-056). The data collection was carried out by double input and through logical verification and sampling inspection of 10% of the input data to control the quality of data collection. If there was a problem with the input data, the researcher would check the original questionnaires in time to ensure the accuracy and reliability of the data.

### Procedures

Before the field investigation, we requested permission from the principals of each school, in order to get the approval and assistance and determine the specific date of the investigation. Once the permissions were granted, investigators conducted the research in each class with the help of the form teachers. Each class is in the charge of the head teacher and two investigators. The purpose of the study as well as the questionnaire sections were explained to students by investigators, and they are required to complete the self-filled questionnaire according to the unified procedures and methods. It takes about 15 min to complete the questionnaire. Students volunteered to participate in the survey, filled in anonymously, and signed the informed consent before the survey. The students were assured of the anonymity and confidentiality of the information provided in the self-reported questionnaires, and the respondents were free to discontinue their participation at any time of the study. After the survey is completed, the investigators will review the questionnaires completed on that day to ensure the quality of the questionnaires.

### Measures

#### Chinese version of Olweus bully/victim questionnaire

The Olweus Bully/Victim Questionnaire (OBVQ) revised by Zhang Wenxin (Hunan Provincial Bureau of Statistics, 2012) is used to investigate traditional bullying and cyber bullying experiences. The Chinese version of OBVQ includes two sub-scales of bullying perpetration and bullying victimization (8 items each), for a total of 16 items. The answer options include “0 = none,” “1 = happened only a few times,” “2 = two or three times a month,” “3 = about once a week,” “4 = several times a week.” Respondents were defined as bullying perpetrators or victims if they answered ≥ 2 on any question in either of the bullying perpetration sub-scales or bullying victimization sub-scales. An answer of ≥ 2 on any question of The Chinese version of OBVQ was defined as participants of school bullying, and others were defined as non-participants. The scale can also be used to assess the frequency of three types of bullying: verbal bullying (swearing, mocking, insulting, threats, etc.), physical bullying (beating, kicking, pushing, etc.), relational bullying (spreading rumors, rejection, neglect, etc.) and cyber bullying. Record the frequency of different types of bullying experienced by the respondents during the semester. The Cronbach’s α coefficients of the two sub-scales were 0.843 and 0.788, respectively.

#### Individual variables

The following variables were selected in the current study based on previous literature (Zhang, 2002; Zhou, 2009; Lam et al., 2013). Personal information includes gender, grade, academic performance, whether transferred to another school in the last 6 months, frequency of playing violent games and the number of social friends. The academic performance of the respondents was classified as good, fair and poor according to their academic ranking in this semester, which is a subjective index. The frequency of playing violent video games only once or twice or playing two or three times a month was classified as occasional, and playing more than once a week was classified as frequent. The number of social friends was classified as no, some and many, by asking the question “how many social friends who do not go to school you have,” which is also a subjective index.

#### Family variables

The survey also collected information at family level based on literature (Zheng et al., 2018; Hellström and Beckman, 2020; Wang et al., 2021), including family structure, family economic status, mother’s level of education, and whether parents or caregivers ever beat their children. Family structure is divided into traditional structure and non-traditional structure, and traditional structure means constituted by a mother and a father. Family economic status refers to the classification of middle school students’ family economic status in a previous study, and this variable is divided into three levels: high, middle and low, which is a subjective indicator. The frequency of describing how often parents beat their children in the last half year coded on a five-point scale, ranging from 1 to 5 (1 = never happened, 2 = only once or twice, 3 = two or three times a month, 4 = about once a week, and 5 = several times a week). And the answer of “two or three times a month,” “about once a week,” “several times a week” were defined as owning the behavior.

#### School variables

School variables included school location, school type and perceived school security, which is a subjective indicator.

### Statistical analysis

All questionnaires were retrieved and coded uniformly, and data were entered using Epidata 3.1(Odense, Denmark). All data were double entered, and automatic comparison checks were performed by using test software. The demographic data of the migrant children and the non-migrant children were compared. The categorical data were analyzed by the chi-squared test. The chi-squared test was used to assess the prevalence of school bullying in the migrant children and the non-migrant children, and the specific variables related to the demographic variables, personal information and parents or caregivers’ behavior were analyzed by the chi-squared test. Statistically significant variables were used in the binary regression analysis. An analysis of the risk factors that may affect the involvement of school bullying in migrant children was conducted. The threshold of significance was defined as *p* < 0.05. The associations were reported *via* adjusted odd ratios (OR) and 95% confidence intervals (95% CI). All statistical analyses were performed using SPSS for Windows 22.0 (IBM, Chicago, IL, United States).

## Results

A total of 7,607 students were surveyed, including 995 migrant children and 6,612 non-migrant children. As shown in Table 1, compared with non-migrant children, migrant children have a higher proportion of boys (*χ*^2^ = 12.804; *p* < 0.001) and senior high school students (*χ*^2^ = 24.635; *p* < 0.001). In terms of family structure, non-traditional families of migrant children account for a larger proportion (*χ*^2^ = 17.930; *p* < 0.001). There are also significant differences in the variables between migrant children and non-migrant children with regard to family economic level, mother’s level of education, school location and school type.

**Table 1:** Descriptive statistics for variables.

Variable	Migrant children (*n* = 995)	Controls (*n* = 6,612)	*χ* ^2^	*p*
**Individual variables**				
Gender			12.804	0.000
Male	525 (52.8%)	3,087 (46.7%)		
Female	470 (47.2%)	3,525 (53.3%)		
Grade			24.635	0.000
Middle school	449 (45.1%)	3,541 (53.6%)		
High school	546 (54.8%)	3,071 (46.4%)		
**Family variables**				
Family structure			17.930	0.000
Traditional	873 (87.7%)	6,070 (91.8%)		
Non-traditional	122 (12.3%)	542 (8.2%)		
Family economic level			9.580	0.000
High	90 (9.0%)	572 (8.7%)		
Middle	742 (74.6%)	5,186 (78.4%)		
Low	163 (16.4)	854 (12.9%)		
Mother’s level of education			11.560	0.009
Elementary school and below	171 (17.2%)	1,007 (15.2%)		
Junior high school	474 (47.6%)	3,239 (49.0%)		
Senior high school or technical secondary school	237 (23.8%)	1781 (26.9%)		
College and upper	114 (11.4%)	585 (8.8%)		
**School variables**				
School location			169.329	0.000
Urban	657 (66.0%)	2,906 (44.0%)		
Rural	338 (34.0%)	3,706 (56.0%)		
School type			25.285	0.000
Public	872 (87.6%)	6,106 (92.3%)		
Private	123 (12.4%)	506 (7.7%)		

Table 2 shows that the involvement rate of school bullying in migrant children was 24.4%, which was higher than the prevalence of 18.1% in non-migrant children (*χ*^2^ = 22.740; *p* < 0.001). And in four forms of school bullying experience, there are significant differences in the involvement rates of migrant children and non-migrant children. The prevalence of verbal, physical, relational and cyber bullying perpetration in migrant children was 6.4, 4.5, 3.8 and 3.5%, which was higher than the prevalence of 3.9% (*χ*^2^ = 14.279; *p* < 0.001), 1.6% (*χ*^2^ = 36.018; *p* < 0.001), 1.4% (*χ*^2^ = 29.743; *p* < 0.001) and 1.0% (*χ*^2^ = 41.902; *p* < 0.001) in non-migrant children, separately. The prevalence of verbal, physical, relational and cyber bullying victimization in migrant children was 17.8, 8.3, 8.7, and 5.5%, which was also higher than the prevalence of 13.3% (*χ*^2^ = 14.289; *p* < 0.001), 4.2% (*χ*^2^ = 31.783; *p* < 0.001), 6.1% (*χ*^2^ = 10.068; *p* = 0.002) and 2.2% (*χ*^2^ = 36.025; *p* < 0.001) in non-migrant children, separately.

**Table 2:** Migrant children and non-migrant children comparison of school bullying.

Variable	Migrant children (*n* = 995)	Controls (*n* = 6,612)	*χ* ^2^	*p*
Involved school bullying			22.740	0.000
Yes	243 (24.4%)	1,195 (18.1%)		
No	752 (75.6%)	5,417 (81.9%)		
Verbal bullying perpetration			14.279	0.000
Yes	64 (6.4%)	255 (3.9%)		
No	931 (93.6%)	6,357 (96.1%)		
Physical bullying perpetration			36.018	0.000
Yes	45 (4.5%)	109 (1.6%)		
No	950 (95.5%)	6,503 (98.4%)		
Relational bullying perpetration			29.743	0.000
Yes	38 (3.8%)	93 (1.4%)		
No	957 (96.2%)	6,519 (98.6%)		
Cyber bullying perpetration			41.902	0.000
Yes	35 (3.5%)	66 (1.0%)		
No	960 (96.5%)	6,546 (99.0%)		
Verbal bullying victimization			14.289	0.000
Yes	177 (17.8%)	882 (13.3%)		
No	818 (82.2%)	5,730 (86.7%)		
Physical bullying victimization			31.783	0.000
Yes	83 (8.3%)	281 (4.2%)		
No	912 (91.7%)	6,331 (95.8%)		
Relational bullying victimization			10.068	0.002
Yes	87 (8.7%)	403 (6.1%)		
No	908 (91.3%)	6,209 (93.9%)		
Cyber bullying victimization			36.025	0.000
Yes	55 (5.5%)	148 (2.2%)		
No	940 (94.5%)	6,464 (97.8%)		

The results in Table 3 show the variables that were significantly different between migrant children involved school bullying (*n* = 243) and those non-involved (*n* = 752). These variables included gender, grade, frequency of playing violent games, transfer to another school in the last 6 months, family economic level, complete family structure, parents or caregivers ever beaten their children, school location and perceived school security. These statistically significant variables were included in the binary logistic regression analysis.

**Table 3:** Risk factors related to school bullying in migrant children (*n* = 995).

Variable	Involved (*n* = 243)	Non-involved (*n* = 752)	*χ* ^2^	*p*
**Individual variables**				
Gender			38.142	0.000
Male	170 (32.4%)	355 (67.6%)		
Female	73 (15.5%)	397 (84.5%)		
Grade			20.248	0.000
Middle school	140 (31.2%)	309 (68.8%)		
High school	103 (18.9%)	443 (81.1%)		
Academic performance			5.501	0.064
Good	76 (21.5%)	277 (78.5%)		
Fair	73 (23.0%)	244 (77.0%)		
Poor	94 (28.9%)	231 (71.1%)		
Transfer to another school in the last six months			6.738	0.009
Yes	27 (37.0%)	46 (63.0%)		
No	216 (23.4%)	706 (76.6%)		
Frequency of playing violent games			43.166	0.000
No	92 (16.7%)	458 (83.3%)		
Occasional	81 (30.7%)	183 (69.3%)		
Frequent	70 (38.7%)	111 (61.3%)		
Numbers of social friends			51.706	0.000
No	78 (15.7%)	418 (84.3%)		
Some	138 (30.9%)	309 (69.1%)		
Many	27 (51.9%)	25 (48.1%)		
**Family variables**				
Family type			4.289	0.038
Traditional	204 (23.4%)	669 (76.6%)		
Non-traditional	39 (32.0%)	83 (68.0%)		
Family economic level			22.856	0.000
High	32 (35.6%)	58 (64.4%)		
Middle	153 (20.6%)	589 (79.4%)		
Low	58 (35.6%)	105 (64.4%)		
Mother’s level of education			2.261	0.520
Elementary school and below	41 (24.0%)	130 (76.0%)		
Junior high school	113 (23.8%)	361 (76.2%)		
Senior high school or technical secondary school	55 (23.2%)	182 (76.8%)		
College and higher	34 (30.1%)	79 (69.9%)		
Parents or caregivers ever beaten their children			33.740	0.000
No	214 (22.6%)	732 (77.4%)		
Yes	29 (59.2%)	20 (40.8%)		
**School variables**				
Perceived school security			24.504	0.000
No	91 (36.0%)	162 (64.0%)		
Yes	152 (20.5%)	590 (79.5%)		
School location			19.653	0.000
Urban	132 (20.1%)	525 (79.9%)		
Rural	111 (32.8%)	227 (67.2%)		
School type			0.441	0.507
Public	210 (24.1%)	662 (75.9%)		
Private	33 (26.8%)	90 (73.2%)		

Table 4 reports the results from binary regression analysis for the entire sample of respondents. Gender and grade are negatively associated with school bullying in migrant children. Being a female and high school student lowers the odds of involving school bullying by 49 and 54%, respectively. More times of playing violent games and numbers of social friends are positively connected to the involvement of school bullying in migrant children. Migrant children who have been beaten by their parents (OR 2.996, 95%CI 1.539–5.831) also influence their school bullying participation.

**Table 4:** Binary regression analysis summary.

Variable	*B*	OR	95%CI	*p*
**Individual variables**				
Gender				
Male		1		
Female	−0.634	0.530	0.374–0.753	0.000
Grade				
Middle school		1		
High school	−0.662	0.516	0.355–0.750	0.001
Transfer to another school in the last 6 months				
No		1		
Yes	0.189	1.208	0.683–2.137	0.516
Frequency of playing violent games				
No		1		0.022
Occasional	0.400	1.492	1.013–2.196	0.043
Frequent	0.571	1.770	1.148–2.729	0.010
Numbers of social friends				
No		1		0.000
Some	0.819	2.269	1.594–3.230	0.000
Many	1.192	3.293	1.680–6.456	0.001
**Family variables**				
Family structure				
Traditional		1		
Non-traditional	−0.169	0.844	0.531–1.344	0.476
Family economic level				
High		1		0.003
Middle	−0.392	0.675	0.401–1.136	0.139
Low	0.300	1.350	0.732–2.489	0.336
Parents or caregivers used to beat their children				
No		1		
Yes	1.097	2.996	1.539–5.831	0.001
**School variables**				
School location				
Rural				
Urban	−0.217	0.805	0.557–1.163	0.248
Perceived school security				
No				
Yes	−0.272			0.132
Constant	−0.827	0.437		0.049

## Discussion

This is the first study to discuss the current situation of school bullying between migrant children and non-migrant children in middle and high schools with such a large and representative sample. Based on the framework of ecological theory, it also marked an initial attempt to explore the influencing factors of school bullying from individual, family and school aspects among migrant children in mainland China.

### Prevalence of school bullying

The rate of involvement of school bullying was 24.4% for migrant children and 18.1% for non-migrant children. Significant differences were found between the two groups, indicating that more school bullying occurred in the migrant group than in the non-migrant group, which was consistent with the results of another cross-sectional study performed in 2019 (Li and Hu, 2020). In addition, the incidence of migrant children was higher than that of non-migrant children in all four forms of bullying: physical bullying, verbal bullying, relational bullying and cyberbullying, and whether it is bullying perpetration or victimization. This is consistent with the findings of Pistella et al. (2020).

Possible explanations for our results are as follows: (1) Under the current hukou system of China, migrants find it difficult to get the same public schools, health care, housing, jobs and insurance as local residents (Li and Gao, 2019). The unfavorable factors, such as the inequality of the system, the restriction of household registration and resettlement, cause a certain degree of pressure on the migrant children. Thus, bullying behavior may be a way for migrant children to cope with stress or to be noticed by others. (2) Migrant children’s socioeconomic status is relatively low, and their parents’ awareness and concept of their rights are still lacking (Wang et al., 2021), which may contribute to their more vulnerable being bullied. (3) Compared with local children, migrant children are an extremely vulnerable population of young people who experience severe hardship in urban settings, and are easily associated with delinquent peers (Liu and Liu, 2016). Because the need for peer acceptance and affiliation is a predictor of bullying and aggressive behavior among immigrants (Strohmeier et al., 2012), this may explain why migrant children have a higher incidence of bullying others.

### Influencing factors associated with school bullying

#### Individual variables

Our study found there are significant gender and grade differences in migrant children’s involvement in school bullying, and being beaten by parents or caregivers is a risk factor of school bullying, these findings are consistent with the results of most previous studies (Hellström and Beckman, 2020; Kennedy, 2021). However, the correlation between violent games and involvement in bullying is controversial. The results of our study found an association between playing violent video games and bullying behavior, and the risk of involvement in bullying increased as the number of violent video games played increased. This is consistent with the results of Liu et al. (2020). A possible explanation is that violent video games are a risk factor for increased aggressive behavior, cognition, and emotion (Anderson et al., 2010), so frequent playing of violent games may increase the risk of involvement in bullying. However, there are still some past studies (Lam et al., 2013; Ferguson et al., 2014a; Ferguson and Olson, 2014b) demonstrated that violent game exposure did not correlate meaningfully with bullying behaviors, nor did it be predictive of bullying. Therefore, our finding deserves further discussion.

Our study also found that the number of social friends was positively associated with involvement in bullying. Previous research has shown that, in theory, both the quality and quantity of friendships can protect against harm. But most previous studies of friends and bullying have not explained the characteristics of the people with whom teens make friends (Mucherah et al., 2018; Shaheen et al., 2019). Our findings suggest that the characteristics of the friends teenagers associate with may influence the extent to which they engage in bullying behavior. This may also be because of the characteristics of migrant children themselves, which encourage them to make more social friends. For example, for children with externalizing behaviors, the more friends they have, the more likely they are to engage in bullying (Duncan, 1999). And adolescents who associate with undesirable peers are also at higher risk of being involved in bullying (Han et al., 2017).

In the regression analysis, the transferring school experience of migrant children had no significant independent predictive effect on school bullying, which does not accord with our hypothesis, the possible reason is that transferring school experience is not one of the most important risk factors for the migrant children, so its role in multiple regression is weakened by other more important factors. However, because this study is the first to explore this question, it is necessary to continue exploring the finding with other samples in the future.

#### Family variables

Consistent with previous studies (Stoltenborgh et al., 2015), this study found that frequent being beaten by parents or caregivers is an important risk factor for school bullying among migrant children, which is in line with the social observation theory or trauma response theory of bullying, that is, children’s bullying behavior is an imitation of their parents’ violent behavior or a traumatic reaction caused by their parents’ beating and scolding (Jia and Mikami, 2014). Furthermore, most previous studies have shown that family economic status affects the social adjustment of migrant children (Shen et al., 2020). These results support the family stress model, which assumes that family economic difficulties affect the stress problems faced by children, and parent–child communication and parental warmth play a moderating role in this connection (Ying et al., 2019). The results of this study did not find a significant correlation between family economy and the involvement in school bullying among migrant children, which may be explained by the lack of research on some possible mitigating factors, so it is needed to further refine our study in the future.

#### School variables

Different from our previous study, in which perceived school security is an important protective for adolescents engaged in school bullying among the general population (Liu et al., 2020), this study did not find a significant correlation between perceived school security and bullying among migrant children. Wen et al. (2009) believed that the sense of perceived security by individuals mainly comes from the social support and the sense of control they can get in life. The migrant children left their hometown and moved to a completely unfamiliar environment, and the perceived security may be limited by the physical and cultural differences between urban and rural areas, as well as by the fact that migrant children live in poor conditions in cities. Therefore, more in-depth studies are needed to investigate the effect of perceived security on school bullying among migrant children.

It is worth mentioning that this study failed to find any school factor that significantly affect school bullying among migrant children, which indicates that in the ecosystem of school bullying, school factors were more remote factors than individual and family factors and the effect of school factors may be weakened by some proximal mediating factors such as teacher-student relationship and class atmosphere. The follow study need to include more school factors or add some mediating factors to further clarify the influence of school factors on school bullying.

### Limitations

Among the limitations of this study was that the self-reported responses used on some questions were not surveyed with mature scales, which was slightly biased; therefore, the results may not be fully generalized. The influencing factors of school bullying among migrant children found in this study need to be further studied. And due to the restrictions of conditions, we cannot get more objective information from the teachers and classmates of migrant children. Moreover, we should also pay more attention to some special identities in migrant children, such as a dual role with both migrant and left-behind, and children who were migrant children before but returned home for various reasons.

## Conclusion

This is the first study to explore the current situation of school bullying among migrant and non-migrant children in middle and high schools with such a large and representative sample. The study found that the prevalence of school bullying among migrant children was 24.4%, higher than that among non-migrant children. Gender, grade, frequency of playing violent games, number of social friends and being beaten by parents or caregivers were significantly correlated with the engagement of migrant children in school bullying. The prevalence of bullying among migrant children indicates that bullying among migrant children has become an important public health problem due to population migration associated with economic development. Our results provide new information on the relationship between migrant population and school bullying, and have practical implications for the intervention of school bullying among Chinese migrant children. Specifically, the prevention of school bullying among migrant children should be targeted at males, middle school students, migrant children who often play violent games and make many social friends and who are often beaten by their parents or caregivers. In addition, migrant children’s caregivers or teachers should provide a more harmonious living and learning atmosphere for migrant children, encourage and educate them to set up good behavior models, reduce bullying behavior, and help migrant children grow up healthily.

## Data availability statement

The raw data supporting the conclusions of this article will be made available by the authors, without undue reservation.

## Ethics statement

The studies involving human participants were reviewed and approved by the ethical committee of Xiangya School of Public Health, Central South University (XYGW-2017-056). Written informed consent to participate in this study was provided by the participants' legal guardian/next of kin.

## Author contributions

ZM-Y: conceptualization. ZM-Y, Y-T, and ZH-Q: visualization, investigation. ZM-Y and Y-T: writing-original draft, data curation. ZM-Y: methodology, formal analysis. XQ-L and DL-L: funding acquisition, resources, supervision, project administration, writing - review and editing. All authors have read and approved the final manuscript.

## Funding

This work was supported by grants from the 13th Five-Year Plan of National Education Science (grant number BBA170061).

## Conflict of interest

The authors declare that the research was conducted in the absence of any commercial or financial relationships that could be construed as a potential conflict of interest.

## Publisher’s note

All claims expressed in this article are solely those of the authors and do not necessarily represent those of their affiliated organizations, or those of the publisher, the editors and the reviewers. Any product that may be evaluated in this article, or claim that may be made by its manufacturer, is not guaranteed or endorsed by the publisher.
